# Preoperative inflammatory biomarkers analysis in prognosis of systemic inflammatory response syndrome following percutaneous nephrolithotomy: A systematic review and meta-analysis

**DOI:** 10.1080/2090598X.2022.2138891

**Published:** 2022-10-31

**Authors:** Dwi Evan Prima Putra Noviardi, Zuhirman, Indra Jaya, Afdal, Joko Pitoyo, Muhammad A. Yashar, Nathanael Ibot David

**Affiliations:** a Department of Surgery, Urology Sub-Division, Faculty of Medicine, Riau University, Pekanbaru, Indonesia; b Faculty of Medicine, Universitas Brawijaya, Malang, Indonesia

**Keywords:** SIRS, leukocyte count, PLR, NLR, CRP

## Abstract

**Introduction:**

Urosepsis is one of the most serious complications of percutaneous nephrolithotomy (PCNL). To date, many studies aim to prescreen urosepsis possibility after PCNL through blood components. This meta-analysis aims to determine C-reactive protein (CRP), neutrophil to lymphocyte ratio (NLR), and platelet to lymphocyte ratio (PLR) obtained preoperatively used to predict postoperative sepsis after PCNL.

**Methods:**

A comprehensive literature search was performed through the electronic databases in March 2022. The quality of the included studies was assessed with Newcastle Ottawa Scale (NOS), while the presence of publication bias was assessed using Begg’s and Egger’s tests. Quantitative analysis was performed using RevMan 5.4 and Comprehensive Meta-Analysis 3.0. The outcome of interest is the difference in blood component count between groups that experienced systemic inflammatory response syndrome (SIRS) and those who did not. Acquired data were pooled as mean difference (MD).

**Results:**

Total of 11 studies were included in the quantitative analysis. Leukocyte count showed an increase between the group that experienced SIRS and those who were not (MD 0.69, 95% confidence interval [CI] 0.48 to 0.91, *p* < 0.00001). Similar result was also found in other analysis, CRP (MD 3.30, 95% [CI] 2.33 to 4.26, *p* < 0.00001), NLR (MD 0.59, 95% [CI] 0.48 to 0.69, *p* < 0.00001), and PLR (MD 23.40, 95% [CI] 17.98 to 28.82, *p* < 0.00001).

**Conclusion:**

Preoperative PLR, NLR, and CRP had significant association with postoperative sepsis after PCNL. It is beneficial for urologists to ensure close monitoring of these biomarkers levels before PCNL. The result of this study might serve as a consideration for future clinical approaches in determining beneficial treatment for urolithiasis patients.

## Introduction

Urolithiasis is the most prevalent major urological condition that accounts for a large number of hospital visits globally [1]. In the United States, renal stones are one of many factors that are responsible for morbidity and the decrease of quality of life, with prevalence of 5% to 10% in their lifetime [2]. Due to its 50% risk for recurrence in a lifetime, it is considered as a recurrent disease [3].

Nowadays, in line with the advances of surgical knowledge, technology, and future aim to reduce the recurrence risk of urolithiasis, minimally invasive techniques are becoming a common treatment option for renal stones [4]. Currently, with the ever-evolving procedure of choice for managing kidney stones, PCNL is more beneficial compared to open surgery due to lower morbidity, shorter convalescence, and reduced cost [5]. Despite its high rate of success, there are complications associated with PCNL. One of the most serious complications following PCNL is urosepsis [6].

**Figure 1.**
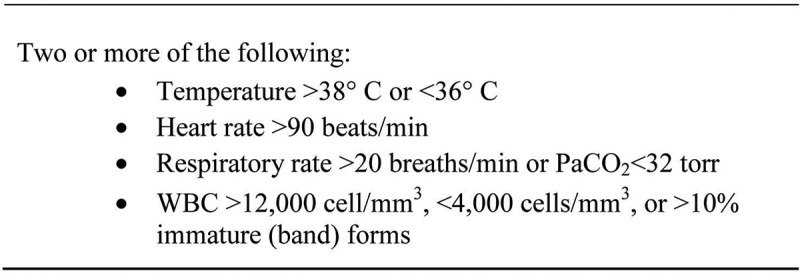
Criteria of systemic inflammatory response syndrome.

After PCNL a major complication like urosepsis (sepsis caused by urinary tract infection) can occure, even with the administration of antibiotic prophylaxis and aseptic urine before surgery. Signs and symptoms of systemic inflammatory response syndrome (SIRS) in patients is essential for the diagnosis of urosepsis [7]. Objectively, SIRS is determined by meeting two of the criteria in Figure 1 [8].

Female gender, presence of urinary diversion, positive preoperative urine culture, preoperative nephrostomy tube, stone size and staghorn stone have been associated with the risk of post-PCNL infection. Unfortunately, all of those have not been consistent across studies and have poor predictive value [9,10]. Thus, there is an urgent need for a pre-operative predictor to identify patients at higher risk, who can then 1) be monitored more intensively or 2) given an additional appropriate antibiotic, which then might be considered whether PCNL is eligible or not.

NLR has been reported that can predict many inflammatory progression and cancerous processes, and several studies have found that the plasma concentrations of proinflammatory cytokines were increased in patients with high NLR. This utility of NLR in predicting inflammatory and malignancies prognosis for patients has been reported by numerous studies [11]. However, NLR’s role to predict infectious complications after PCNL has not yet been investigated.

The platelet-to-lymphocyte ratio (PLR) is another inflammatory factor that has been reported as a promising biomarker in predicting malignancy prognosis. However, its use in urosepsis conditions has been investigated in limited studies. Studies showed that pre-operatively elevated NLR and PLR could be used as markers for fever and SIRS after PCNL [7,12]. Prevention to reduce the risk of SIRS after PCNL can be made by using these markers.

Despite the limited use in the early diagnosis of bacterial infections, C-reactive protein, leukocyte count, are the most commonly used parameters. It has been previously found that NLR was a sensitive marker to predict post-PCNL fever in patients [13]. The retrospective study of patients who underwent PCNL over a one-year period found that a preoperative CRP was able to predict the development of postoperative SIRS. CRP is a cytokine-mediated inflammation marker and a sensitive acute phase reactant. To date, there is no strong evidence to support its use before surgery, and its role in urolithiasis has not been investigated in previous studies.

Predicting SIRS, which is associated with sepsis and other complications, is important for both the physicians and patients. In this study, we aimed to determine whether leukocyte count, NLR, PLR, and CRP obtained from routine preoperative blood tests can be used to predict postoperative sepsis after PCNL in patients with renal stones.

## Material and methods

### Study design

This systematic review was performed through several online databases such as ScienceDirect, Cochrane Library, Google Scholar, PubMed, and ProQuest and completed in March 2022. This comprehensive study was carried out according to Preferred Reporting Items for Systematic Reviews and Meta-analyses (PRISMA) guidelines. We conducted a literature search on the comparison of systemic inflammatory response syndrome (SIRS) event and no SIRS event on urolithiasis patients after PCNL. Studies that are included in the quality assessment of the study have fulfilled the predetermined eligibility criteria. Important information from the studies based on the results of study risk of bias quality assessment was extracted. The exposure variable was PCNL approach and then we divided the patients into SIRS group and no SIRS group. The primary outcome of this study was the relationship between leukocyte count and SIRS event postoperative. The secondary outcome included the relationship of: (1) C-Reactive Protein (CRP), (2) Neutrophil to Lymphocyte Ratio (NLR), and (3) Platelet to Lymphocyte Ratio (PLR) and SIRS event postoperative. The pooled mean difference (MD) and 95% confidence interval (CI) were applied in determining the overall event.

### Search strategy

**Figure 2.**
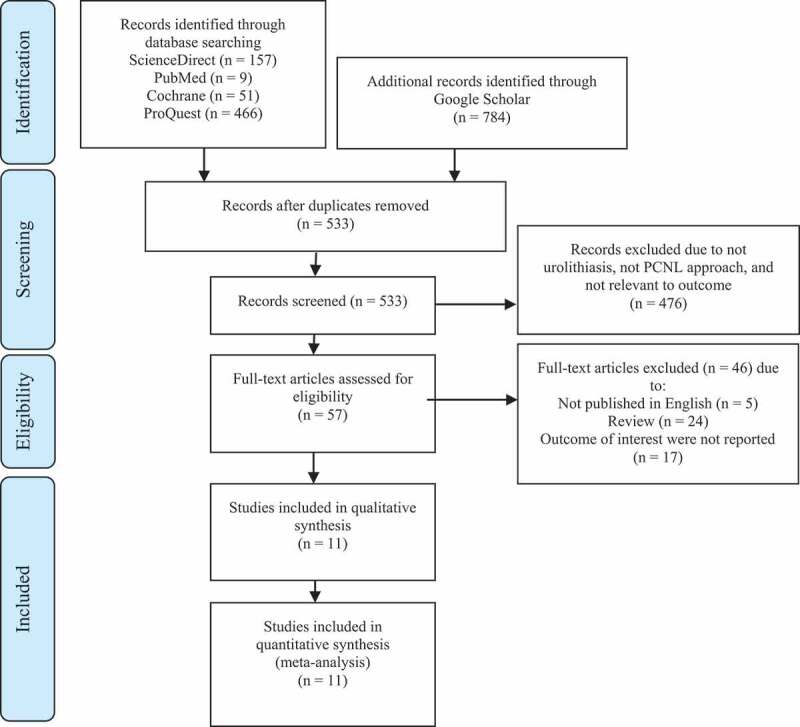
PRISMA flowchart of the screening criteria and included studies.

A comprehensive literature search of studies that discuss the correlation of PCNL approach towards the event of SIRS was performed by the author until March 2022. We used several keywords on the search of study such as ‘percutaneous nephrolithotomy’ or ‘PCNL’, AND ‘predictive’ or ‘predictive value’ or ‘predictive factor’ or ‘risk factor’, AND ‘systemic inflammatory response syndrome’ or ‘SIRS’, AND ‘leukocyte’ or ‘White Blood Cell’ or ‘WBC’, AND ‘C-Reactive Protein’ or ‘CRP’, AND ‘neutrophil to lymphocyte ratio’ or ‘NLR’, AND ‘platelet to lymphocyte ratio’ or ‘PLR’. Two reviewers (Noviardi DEPP and David NI) performed and cross-checked the search and selection of literature independently. Disagreements between the reviewers that arise during the writing process were resolved by a discussion. Total of the included literature that has been screened is shown in Figure 2.

### Eligibility criteria

Consideration on entering the appropriate studies were made based on the following criteria: All accessible studies which investigated the occurrence of SIRS or its progression from fever, SIRS, to sepsis; studies with purpose to investigate predictive value or prognostic value of preoperative inflammatory biomarkers in complication of SIRS after PCNL; article about PCNL complications related to progression of fever, SIRS, and sepsis; and studies that published within last five years. The evaluated outcomes should include leukocyte count, C-Reactive Protein (CRP), neutrophil to lymphocyte ratio (NLR), and platelet to lymphocyte ratio (PLR). On the other hand, studies were excluded if they have inaccessible full texts, published other than using English, irrelevant outcomes, or low-quality studies (high risk of bias).

### Data collection and statistical analysis

All the included studies were assessed manually for duplication and in accordance with the determined eligibility criteria. The quality of included studies was assessed using the Newcastle-Ottawa Scale (NOS) for Retrospective Study. The formulas developed by [14,15] were utilized to calculate the missing mean and standard deviation (SD) of studies that displayed continuous outcome using median and range values, and median and interquartile range (IQR) values. Heterogeneity of the studies was evaluated by *I^2^* statistics. A cut-off *I^2^* value of more than 50% was used to determine the significant presence of heterogeneity. An analysis using a fixed-effect model was used if the heterogeneity was insignificant. A *p-*value of <0.05 in the difference between SIRS and No SIRS group was considered to be as statistically significant. Publication bias was considered when the *p-*value of Egger’s and Begg’s test <0.05. The overall quantitative analysis was performed using a combination of two statistical software Comprehensive Meta-Analysis version 3.0 (CMA, New Jersey, USA) and Review Manager version 5.4 (RevMan, Cochrane Collaboration, Oxford, United Kingdom).

### Outcomes

In this study the primary outcomes evaluated the change difference of estimated leukocyte count, CRP, NLR, and PLR between preoperative and postoperative on groups that experienced SIRS event and those who don’t experienced it. Data were pooled as mean difference (MD) for continuous data.

## Results

Studies that are included in this meta-analysis started with 533 screened titles and abstracts, for full-text eligibility 57 studies were accessed. Then 11 were included for meta-analysis. A search flow diagram is presented in Figure 1. Three studies were conducted based on multi-center data [12,16,17], while the other study collected from single-center [9,18–24]. Regarding the variable used to determine the outcome of this paper, eight studies provide the data for leukocyte count [9,16–18,20,22–24]. Three studies provide the data for CRP [9,19,20], six studies provide the data for NLR [12,17,21], and five studies provide the data for PLR [12,18–21].

Summary of the included studies consists of country, data source, outcome, study design, enrolment period, number of participants, and NOS quality assessment are presented in Table 1, while the bias assessment is displayed in Table 2.

**Table 1.** Characteristic of the participant in this study.

No.	Author	Country	Data Source	Outcome	Enrolment Period	Participant			Newcastle-Ottawa Scale (NOS)
						Total	SIRS (*n*)	No SIRS (*n*)	
1.	Xu H., 2019	China	The First Affiliated Hospital of Soochow University	WBC,NLR,PLR	August 2015 to February 2018	556	123	433	9
2.	Ganesan V.,2017	United States of America	Cleaveland Clinic	WBC, CRP	October 2012 to October 2013	107	35	72	7
3.	Cetinkaya M.,2017	Türkiye	Mugla Sitki Kocman University, Medova Hospital, Namik Kemal University, and Samsun Education and Research Hospital	NLR, PLR	2013 to 2015	192	41	151	9
4.	Akdeniz E., 2021	Türkiye	Gazi Hospital	CRP, NLR, PLR	January 2015 to January 2020	228	29	199	8
5.	Qi T., 2021	China	Shandong Provincial Hospital Affiliated to Shandong First Medical University, Cheeloo College of Medicine, and Qingyun County People Hospital	WBC	April 2019 to September 2019	90	16	74	9
6.	Tang Y., 2021	China	The First Affiliated Hospital of Sun Yat-sen University and The Third Affiliated Hospital of Sun Yat-sen University	WBC, NLR	January 2016 to December 2020	758	97	661	8
7.	Peng C., 2021	China	Shaoxing People’s Hospital	WBC,CRP,NLR,PLR	May 2016 to May 2020	365	108	257	8
8.	Kriplani A.,2022	India	Tertiary Referral Center, Karnataka, India	NLR, PLR	November 2018 toOctober 2019	517	56	461	8
9.	Liu J.,2021	China	Peking University People’s Hospital	WBC	June 2012 to December 2019	241	41	200	7
10.	Chan J.Y.H.,2021	Canada	University of British Columbia	WBC	2009 to 2015	99	40	59	9
11.	Amri M.,2019	Tunisia	Hospital Ibn El Jazzar, Tunisia	WBC	January 2012 to January 2016	170	34	136	7

**Table 2.** The result of inflammatory biomarkers in SIRS and NO SIRS patients.

No.	Outcome	Participant		Model	Mean Difference	95% CI		*P* Value of Heterogeneity	*P* Value of Begg’s Test	*P* Value of Egger’s Test	*P*
		SIRS (n)	NO SIRS (n)			Lower Limit	Upper Limit				
1.	C-Reactive Protein (CRP)	172	528	Fixed	2.45	2.06	5.19	0.11	0.29	0.50	<0.00001
2.	Neutrophil to Lymphocyte Ratio (NLR)	454	2162	Fixed	0.59	0.48	0.69	0.41	1.00	0.82	<0.00001
3.	Platelet to Lymphocyte Ratio (PLR)	357	1501	Fixed	23.40	17.98	28.82	0.38	0.46	0.25	<0.00001
4.	White Blood Cells (WBC)	492	1894	Fixed	0.69	0.48	0.91	0.07	0.06	0.18	<0.00001

### Primary outcome

#### Leukocyte count

**Figure 3.**
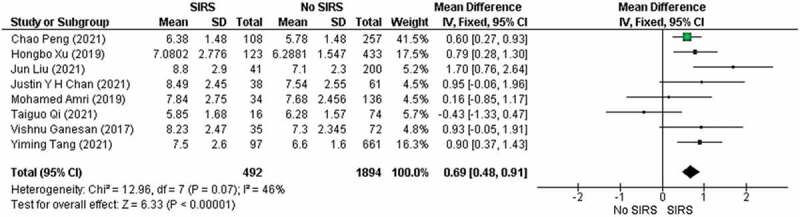
Forest plot for preoperative leukocyte count in patients developed with SIRS and no SIRS. SD, standard deviation; IV, inverse variance; CI, confidence interval.

Total of 2386 participants that were evaluated from eleven studies were allocated into SIRS (*n* = 492) and No SIRS groups (*n* = 1894) to analyze the leukocyte count of both groups. In Figure 3 pooled analysis showed that the studies included had a significant increase between the group that experienced SIRS and those who were not (MD 0.69, 95% [CI] 0.48 to 0.91, *p* < 0.00001). Moderate heterogeneity was apparent between all qualified studies, with an (*I^2^ *= 46% and *p* heterogeneity = 0.07).

In subgroup analysis evaluating country and study design in Table 3 group A the pooled MD showed a significant increase between the group that experienced SIRS and those who were not in People’s Republic of China, United States of America and retrospective subgroup analysis. Analysis result on the Republic of Tunisia subgroup showed an insignificant increase between the group that experienced SIRS and those who were not.

**Table 3.** Result of subgroup analyses (A) white blood cell count (B) C-reactive protein (C) neutrophil to lymphocyte ratio (D) platelet to lymphocyte ratio.

	Study	Participant	Mean Difference (95% CI)	p-value	p-value of heterogeneity	*I^2^*(%)
**A**						
Overall Result	8	2386	0.69 (0.48 to 0.91)	<0.00001	0.07	46%
Country						
People’s Republic of China	5	2010	0.69 (0.46 to 0.92)	<0.00001	0.02	65%
United States of America	2	206	0.94 (0.24 to 1.64)	0.009	0.98	0%
Republic of Tunisia	1	170	0.16 (−0.85 to 1.17)	0.76	NA	NA
Study Design						
Retrospective	8	2386	0.69 (0.48 to 0.91)	<0.00001	0.07	46%
B						
Overall Result	3	700	3.30 (2.33 to 4.26)	<0.00001	0.11	54%
Country						
People’s Republic of China	1	365	2.45 (1.19 to 3.71)	<0.00001	NA	NA
Republic of Türkiye	1	228	4.38 (2.48 to 6.28)	<0.00001	NA	NA
United States of America	1	107	4.80 (2.26 to 7.34)	<0.00001	NA	NA
Study Design						
Retrospective	3	700	3.30 (2.33 to 4.26)	<0.00001	0.11	54%
C						
Overall Result	6	2616	0.59 (0.48 to 0.69)	<0.00001	0.41	0%
Country						
People’s Republic of China	3	1679	0.57 (0.46 to 0.68)	<0.00001	0.34	7%
Republic of Türkiye	2	420	0.57 (0.21 to 0.92)	0.002	0.61	0%
Republic of India	1	517	1.10 (0.46 to 1.74)	0.0007	NA	NA
Study Design						
Retrospective	5	2099	0.57 (0.47 to 0.68)	<0.00001	0.66	0%
Prospective	1	517	1.10 (0.46 to 1.74)	0.0007	NA	NA
D						
Overall Result	5	1858	23.40 (17.98 to 28.82)	<0.00001	0.38	4%
Country						
People’s Republic of China	2	921	21.45 (14.25 to 28.65)	<0.00001	0.87	0%
Republic of Türkiye	2	420	30.76 (21.02 to 40.51)	<0.00001	0.64	0%
Republic of India	1	517	13.90 (−1.53 to 29.33)	0.08	NA	NA
Study Design						
Retrospective	4	1341	24.74 (18.95 to 30.53)	<0.00001	0.47	0%
Prospective	1	517	13.90 (−1.53 to 29.33)	0.08	NA	NA

CI, confidence interval; NA, Not Applicable

### Secondary outcome

#### C-reactive protein (CRP)

**Figure 4.**
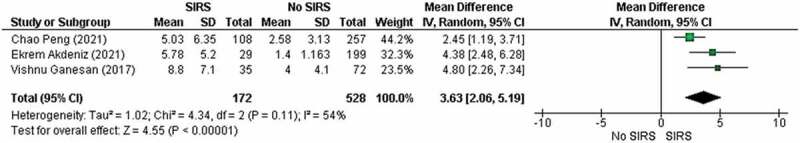
Forest plot for preoperative C-reactive Protein in patients developed with SIRS and no SIRS. SD, standard deviation; IV, inverse variance; CI, confidence interval.

Total of 700 participants that were evaluated from three studies were allocated into SIRS (*n* = 172) and No SIRS groups (*n* = 528) to analyze the CRP of both groups. In Figure 4 pooled analysis showed that the studies included had a significant increase between the group that experienced SIRS and those who were not (MD 3.30, 95% [CI] 2.33 to 4.26, *p* < 0.00001). Moderate heterogeneity was apparent between the qualified studies, with an (*I^2^ *= 54% and *p* heterogeneity = 0.11).

In subgroup analysis evaluating country and study design in Table 3 group B the pooled MD showed a significant increase between the group that experienced SIRS and those who were not in People’s Republic of China, Republic of Türkiye, United States of America, and in retrospective subgroup analysis.

#### Neutrophil to lymphocyte ratio (NLR)

**Figure 5.**
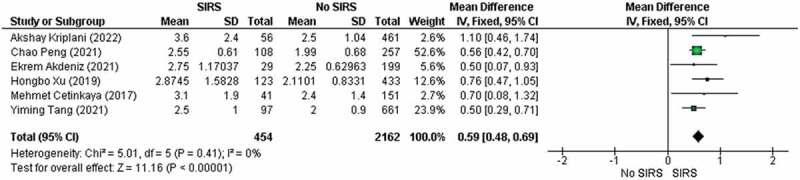
Forest plot for preoperative neutrophil to lymphocyte ratio in patients developed with SIRS and no SIRS. SD, standard deviation; IV, inverse variance; CI, confidence interval.

Total of 2616 participants that was evaluated from six studies were allocated into SIRS (*n* = 454) and No SIRS groups (*n* = 2162) to analyze the NLR of both groups. In Figure 5 pooled analysis showed that the studies included had a significant increase between the group that experienced SIRS and those who were not (MD 0.59, 95% [CI] 0.48 to 0.69, *p* < 0.00001). Low heterogeneity was apparent between the qualified studies, with an (*I^2^ *= 0% and *p* heterogeneity = 0.41).

In subgroup analysis based on country, study design in Table 3 group C the pooled MD showed a significant increase between the group that experienced SIRS and those who were not in People’s Republic of China, Republic of Türkiye, Republic of India, retrospective study, and prospective study subgroup.

#### Platelet to lymphocyte ratio (PLR)

**Figure 6.**
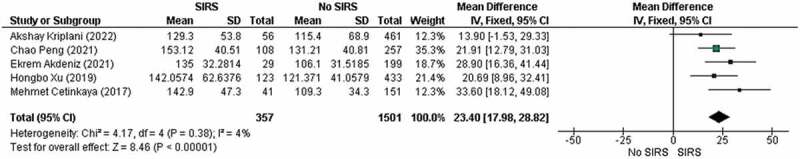
Forest plot for preoperative platelet to lymphocyte ratio in patients developed with SIRS and no SIRS. SD, standard deviation; IV, inverse variance; CI, confidence interval.

Total of 1858 participants that were evaluated from five studies were allocated into SIRS (*n* = 357) and No SIRS groups (*n* = 1501) to analyze the PLR of both groups. In Figure 6 pooled analysis showed that the studies included had a significant increase between the group that experienced SIRS and those who were not (MD 23.40, 95% [CI] 17.98 to 28.82, *p* < 0.00001). Low heterogeneity was apparent between the qualified studies, with an (*I^2^ *= 4% and *p* heterogeneity = 0.87).

In subgroup analysis based on country, study design in Table 3 group D the pooled MD showed a significant increase between the group that experienced SIRS and those who were not in People’s Republic of China, Republic of Türkiye, and retrospective study subgroup. Republic of India and prospective study subgroup result showed an insignificant increase between the group that experienced SIRS and those who were not.

### Association between sex and stone location to the risk of SIRS

Subgroup analyses consisted of sex and stone type location showed the risk of SIRS in each group. The result in Table 4 showed female patients had a higher risk of SIRS after PCNL compared to male patients (OR 1.83, 95% [CI] 1.52 to 2.21, *p* < 0.00001, *I^2^ *= 1%). In the stone type location group, the result showed staghorn stone associated with higher risk of sepsis compared to other locations (OR 2.52, 95% [CI] 1.76 to 3.60, *p* < 0.00001, *I^2^ *= 0%).

**Table 4.** Result of sex and stone type location subgroup analyses.

	Study	Participant	Odds Ratio (95% CI)	p-value	p-value of heterogeneity	*I^2^*(%)
Sex						
Male	11	2071	0.55 (0.45 to 0.66)	<0.00001	0.44	1%
Female	11	1252	1.83 (1.52 to 2.21)	<0.00001	0.44	1%
Stone Type						
Pelvis	2	420	0.84 (0.45 to 1.58)	0.59	0.36	0%
Calyx	2	420	0.96 (0.25 to 3.66)	0.95	0.05	75%
Pelvis + Calyx	2	420	0.71 (0.39 to 1.28)	0.26	0.58	0%
Staghorn	5	1348	2.52 (1.76 to 3.60)	<0.00001	0.41	0%

CI, confidence interval; NA, Not Applicable

## Discussion

This study involved 11 studies on the use of PCNL for meta-analysis. New studies that have not been analyzed in previous meta-analysis were added and that resulted in interesting new insights to the topic. Nowadays, in line with the advances of surgical knowledge and technology, which focuses on the use of minimally invasive techniques, PCNL is becoming a common treatment option for renal stones [4].

This study showed that Leukocyte Count, CRP, Neutrophil to Lymphocyte Ratio, and Platelet to Lymphocyte Ratio had a significant increase between the group that experienced SIRS and those who were not. We conducted subgroup analysis by country, study design, gender, and stone location to identify association between each subgroup and the risk of SIRS after PCNL, and any potential factors from the result that may affect the heterogeneity level between the studies. In subgroup analyses through our meta-analyses, a significant association of preoperative leukocyte count, CRP, NLR, and PLR with postoperative sepsis in patients who undergo PCNL was reported in almost all of the included studies. Result of sex and stone type location risk of SIRS were highly associated with female and staghorn stone compared to male and other stone location. Also it may explain the association with heterogeneity of the result because of the differences of patients’ characteristics.

### Primary outcome

#### Leukocyte count

In this study, increase of Leukocyte Count is a significant predictor for patient developed with SIRS. Some studies suggest leukocyte count has a high sensitivity and specificity as a predictor of urosepsis. It has been reported that the presence of elevated leukocyte count was an independent risk factor for postoperative urosepsis secondary to PCNL [25]. Higher level of serum leukocyte was more likely to be detected in female patients with SIRS than in male patients with SIRS [20]. Other studies in Türkiye showed that univariate and multivariate analysis revealed that higher preoperative leukocyte count statistically significant parameters related with post-PCNL [13]. However, this result is different from the cross-sectional meta-analysis conducted by Jang which stated that preoperative Leukocyte Count had no significant relationship with SIRS status [26].

### Secondary outcome

#### C-reactive protein (CRP)

C-reactive protein (CRP) is a sensitive acute phase reactant that is frequently used as a clinical indicator marker of systemic inflammation [27]. This study was in accordance with nine studies of meta-analysis in which a moderate degree of the overall area under the summary receiver operator characteristic (SROC) curve was found for the diagnostic accuracy of CRP for SIRS in adult patients [28]. The univariate analysis revealed that CRP is an independent risk factor for postoperative SIRS after PCNL with a statistically significant difference between groups in preoperative serum CRP >3.16 mg/L (*p* < 0.001) [19]. However, this study was different from previous research which stated that CRP is not a significant predictor for postoperative SIRS (*p* = 0.011) [29].

#### Neutrophil to lymphocyte ratio (NLR)

Neutrophil to lymphocyte ratio is often used as a predictor of SIRS and has shown a significant correlation. It is a cheaper and rapidly available marker than the other existing markers of inflammation and SIRS [30]. In healthy people (without differences in race or sex category) mean NLR value is below 2, and with good sensibility and specificity in sepsis it may increase up to the values of >10 and >20 in septic shock [31].

The pooled analysis of NLR in nine studies (1371 patients) showed significantly higher NLR in SIRS than in non-SIRS patients. This meta-analysis indicates that higher NLR values may indicate unfavorable prognoses in these patients [32]. Other studies also reported similar findings regarding the higher NLR values in critically ill group (*p* < 0.05) and nephrolithiasis patients which demonstrate the effectiveness of NLR in predicting possible post-PCNL SIRS [32,33].

#### Platelet to lymphocyte ratio (PLR)

A study of 192 renal stone patients showed that SIRS developed postoperatively in 41 (21.3%) patients who had undergone conventional PCNL. Significant difference was revealed in univariate analysis of preoperative PLR (*p* < 0.001) while in multivariate analysis only PLR showed as one of the independent factors that affect the SIRS development. SIRS development was predicted when the PLR cut-off value was 114.1, with 80.4% in sensitivity and 60.2% in specificity [12]. Other study in Turkey with 517 patients evaluated showed that postoperative SIRS had significant association with PLR that can be useful as a cost-effective, easily accessible, and independent predictor for early identification of post-PCNL SIRS/sepsis [21]. A similar retrospective study of 756 patients that underwent PCNL for renal stones between 2012 and 2019 revealed a significant association in the univariate and multivariate analysis between preoperative PLR and the presence of SIRS (*p* < 0.001) with 81% in sensitivity and 80.1% in specificity when the cut-off value of PLR was 120.5 [34].

### Strengths and limitations

This study has a number of strengths. This meta-analysis was the first that explored the association between preoperative inflammatory biomarkers value and the risk of SIRS after PCNL surgery. The review used a holistic approach, with evaluating three indicators like, CRP, NLR, and PLR to measure the risk of postoperative SIRS after PCNL while also included the review of white blood cell count as the primary indicator of SIRS. Study methodology characteristics were performed to stratificate the subgroup (i.e. countries and study design) to determine if these variables have an association and affect the level of heterogeneity of this meta-analysis while minimizing other relevant factors that may influence the overall results according to the PRISMA guidelines. Other than that, this study also add subgroup of sex and stone type location to see their association with risk of SIRS in all of the study included. Robust statistical procedures were used, and all of the information extracted from high-quality studies was based on the results of the study risk of bias quality assessment.

This study also has several limitations. First, almost all of the studies included are retrospective studies which have disadvantages such as risk of bias and potential missing data. Second, the comparability of findings across the included studies may vary because of the wide variability in age, operation time, stone location, stone size, body mass index, and the difference laboratory result interval of each ethnics/race [35]. Third, the average values obtained from each study were not the same for each indicator and had a fairly high standard deviation. Because all of the studies that included were obtained from different countries, and the variability of patient, past clinical and surgery history, and the difference in interval value of each indicator from each race/ethnic made the result obtained had moderate heterogeneity [36]. Finally, although subgroup analysis showed that the stability of the results had no significant change, more high quality and multicenter research need to be performed to verify the results of the study. This study couldn’t determine the baseline value of PLR, NLR, and CRP preoperatively to predict SIRS, but to show these indicators that obtained preoperatively can be helpful for physicians to reinforce clinical consideration of SIRS.

## Conclusion

Since PLR, NLR, and CRP are significant predictors in prescreening of urosepsis after PCNL, these variables could support Leukocyte count as Biomarker indicator for SIRS. These other blood components are not to replace leukocyte count as primary indicator for SIRS. But as supportive markers for physicians to reinforce clinical consideration of SIRS and urosepsis. It is beneficial for urologists to ensure close monitoring of these biomarkers levels before PCNL. The result of this study might serve as a consideration for future clinical approaches in determining beneficial treatment for urolithiasis patients.

## Figures and Tables

**Table:** 

PCNL	Percutaneous Nephrolithotomy
CRP	C-reactive protein
NLR	Neutrophil to Lymphocyte Ratio
PLR	Platelet to Lymphocyte Ratio
NOS	Newcastle-Ottawa Scale
SIRS	Systemic Inflammatory Response Syndrome
MD	Mean Difference
CI	Confidence Interval
WBC	White Blood Cell
SD	Standard Deviation
IQR	Interquartile Range
TF	Tissue Factor
NETs	Neutrophil Extracellular Traps
